# Screening for Cognitive Impairment After Stroke: Validation of the Chinese Version of the Quick Mild Cognitive Impairment Screen

**DOI:** 10.3389/fneur.2021.608188

**Published:** 2021-03-05

**Authors:** Yangfan Xu, Lingrong Yi, Yangyang Lin, Suiying Peng, Weiming Wang, Wujian Lin, Peize Chen, Weichao Zhang, Yujie Deng, Suimin Guo, Le Shi, Yuling Wang, D. William Molloy, Rónán O'Caoimh

**Affiliations:** ^1^Department of Rehabilitation Medicine, The Sixth Affiliated Hospital, Sun Yat-sen University, Guangzhou, China; ^2^Department of Rehabilitation Medicine, The Second Affiliated Hospital, Chongqing Medical University, Chongqing, China; ^3^Center for Child Health and Mental Health, Shenzhen Children's Hospital, Shenzhen, China; ^4^Centre for Gerontology and Rehabilitation, University College Cork, St Finbarr's Hospital, Cork, Ireland; ^5^Department of Geriatric Medicine, Mercy University Hospital, Cork, Ireland; ^6^Clinical Sciences Institute, National University of Ireland Galway, Galway, Ireland

**Keywords:** cognition screen, mild cognitive impairment, post-stroke dementia, China, stroke, Q*mci*-CN

## Abstract

**Background:** Screening for post-stroke cognitive impairment (PSCI) is necessary because stroke increases the incidence of and accelerates premorbid cognitive decline. The Quick Mild Cognitive Impairment (Q*mci*) screen is a short, reliable and accurate cognitive screening instrument but is not yet validated in PSCI. We compared the diagnostic accuracy of a Chinese version of the Q*mci* screen (Q*mci*-CN) compared with the widely-used Chinese versions of the Montreal Cognitive Assessment (MoCA-CN) and Mini-Mental State Examination (MMSE-CN).

**Methods:** We recruited 34 patients who had recovered from a stroke in rehabilitation unit clinics in 2 university hospitals in China: 11 with post-stroke dementia (PSD), 15 with post-stroke cognitive impairment no dementia (PSCIND), and 8 with normal cognition (NC). Classification was made based on clinician assessment supported by a neuropsychological battery, independent of the screening test scores. The Q*mci*-CN, MoCA-CN, and MMSE-CN screens were administered randomly by a trained rater, blind to the diagnosis.

**Results:** The mean age of the sample was 63 ± 13 years and 61.8% were male. The Q*mci*-CN had statistically similar diagnostic accuracy in differentiating PSD from NC, an area under the curve (AUC) of 0.94 compared to 0.99 for the MoCA-CN (*p* = 0.237) and 0.99 for the MMSE-CN (*p* = 0.293). The Q*mci*-CN (AUC 0.91), MoCA-CN (AUC 0.94), and MMSE-CN (AUC 0.79) also had statistically similar accuracy in separating PSD from PSCIND. The MoCA-CN more accurately distinguished between PSCIND and normal cognition than the Q*mci*-CN (*p* = 0.015). Compared to the MoCA-CN, the administration times of the Q*mci*-CN (329s vs. 611s, respectively, *p* < 0.0001) and MMSE-CN (280 vs. 611s, respectively, *p* < 0.0001) were significantly shorter.

**Conclusion:** The Q*mci*-CN is accurate in identifying PSD and separating PSD from PSCIND in patients post-stroke following rehabilitation and is comparable to the widely-used MoCA-CN, albeit with a significantly shorter administration time. The Q*mci*-CN had relatively poor accuracy in identifying PSCIND from NC and hence may lack accuracy for certain subgroups. However, given the small sample size, the study is under-powered to show superiority of one instrument over another. Further study is needed to confirm these findings in a larger sample size and in other settings (countries and languages).

## Introduction

Post-stroke cognitive impairment (PSCI) is increasingly prevalent among community-dwelling older adults, due to the increasing incidence and the decreasing mortality associated with stroke (1, 2). This trend is also evident in China (3). According to the degree of cognitive decline and impairment in activities of daily living (ADL), patients with PSCI can be divided into 2 groups: post-stroke cognitive impairment no dementia (PSCIND), those with slight cognitive impairment with or without subtle impairment in instrumental ADL (IADL) and those with established post-stroke dementia (PSD) with definite impairment in basic ADL (BADL) (4). PSCI is common in China, affecting ~80% of stroke survivors of which PSCIND and PSD represent around 49% and 32% of cases, respectively (5). Once established cognition may decline rapidly (6). Despite this, care usually focuses on post-stroke physical disability with less attention paid to cognitive decline (7–9). PSCI has a significant impact on independence and on the potential to return to work after stroke (10). Given these points, international guidelines recommend cognitive screening for those at risk of PSCI (4, 11).

As yet, no single screening and assessment tool is recommended, although tools should be selected according to the population being evaluated, the stage of progression based on functional status, the preferences of patients and families, as well as the resources available (4). Studies show that both the Montreal Cognitive Assessment (MoCA) and Mini Mental State Examination (MMSE) are both widely-used to detect cognitive impairment after stroke, though in a recent systematic review comparing the accuracy and utility of instruments, the MoCA was shown to be the most valid and clinically feasible to identify PSCI (12). The Chinese version of the MoCA (MoCA-CN) shows high sensitivity and specificity for PSCI (13) as well as other types of vascular cognitive impairment (14), although the Chinese version of MMSE (MMSE-CN) remains the most widely-used screening instrument in China (4).

The Quick mild cognitive impairment (Q*mci*) screen is a new, short cognitive screening instrument originally designed to identify mild cognitive impairment (MCI), which is highly accurate in differentiating between normal cognitive function, subjective cognitive disorders, MCI and early dementia (15, 16). The Q*mci* screen is more accurate than MMSE in detecting early cognitive changes (17–19) and when compared to the MoCA, it has similar accuracy and better specificity but a shorter administration time (20–22). The Chinese version of the Q*mci* (Q*mci*-CN) has recently been validated in a Chinese general rehabilitation outpatient clinic sample (23). To date, the Q*mci* screen has not been examined in patients post-stroke. Given this point, the aim of this research was to investigate the diagnostic accuracy of the Q*mci*-CN in detecting cognitive impairment by comparing it to Chinese versions of the standardized MMSE (MMSE-CN) and MoCA (MoCA-CN) in patients recovering from stroke attending Chinese rehabilitation unit clinics.

## Methods

### Participants

Consecutive participants were recruited by therapists from rehabilitation clinics in two hospitals in Guangzhou, China between August 2017 and April 2018. All patients were aged over 18 years of age and had a stroke diagnosed by a neurologist confirmed with brain imagining (CT or MRI). Only patients consented by their attending doctor participated. The study excluded patients who were unable to communicate verbally in Chinese or had communication problems that could influence the performance of cognitive testing such as severe dysphasia, apraxia, visual impairment, hearing loss, or altered consciousness. Patients with known dementia prior to the stroke or those with existing neurological, psychiatric disorders or other comorbidities that potentially affect cognitive function, e.g., Parkinson's disease were also excluded. Further, patients who couldn't read and write, those with a recent history of alcohol or drug abuse and those who declined to participate were excluded. All those included signed informed written consent. This study received ethical approval from The Six Affiliated Hospital of Sun Yat-sen University, reference number 2019ZSLYEC-110.

### The Q*mci*-CN

The Q*mci* screen has 6 subtests covering 5 cognitive domains (16). The first subtest examines orientation to place and time. The second, five-word registration, examines attention and working memory. The third, a clock drawing test, examines visuospatial/executive function and attention. The fourth subtest is delayed recall examining episodic memory (5-word recall). The next subtest is categorical verbal fluency tests sematic memory and language (e.g., naming of animals in 1 min). The final subtest is logical memory, immediate verbal recall of a short story, which also examines episodic memory. Each subtest is time-limited with an administration time of ~5 min for the Q*mci* screen (16). The total possible score is 100 points with higher scores suggesting normal cognition. The optimal cut-off score for cognitive impairment (MCI and dementia vs. normal controls) for the Q*mci* screen (original English language version) is ≤62/100 (20). However, the optimal cut-off for the Chinese version (Q*mci*-CN) is lower at ≤55/100 (23). This difference is considered to be multi-factorial but most likely relates to the small sample of Chinese patients included in the single validation study to date (23).

### Data Collection

Patients' characteristics including their age, sex, educational level, and other demographic and clinical information were collected. The Q*mci*-CN, MoCA-CN, and MMSE-CN were administered in random order by a trained rater, who was blind to the diagnosis. The interval between administration of the scales was ~5 min. Following screening, patients were administered a battery of neuropsychologist tests and the Lawton Instrumental Activities of Daily Living (IADL) scale (24) scale to support a clinician to make a diagnosis and classify patients into those with normal cognition, PSCIND, and PSD. The diagnostic classification was made independent of the results of the cognitive screening instruments. The following neuropsychological tests were used to assess cognitive function: (1) The Auditory Verbal Learning Test–H tested memory including immediate and delayed recall (25); (2) The Rey-Osterrieth Complex Figure Test (RCFT) was used to examine the visuospatial function domain (26, 27); (3) Animal Fluency Test was used to assess language (27);(4) and the Chinese modified version of the Trail Making Test (TMT-A, TMT-B) was used to examine executive function (28). The caregiver burden inventory was also administered to the family or nurse of the patient to assess carer strain (29). These widely-used scales were chosen as they have good reliability and validity. PSCIND was diagnosed among those with decline in at least 1 cognitive domain (memory, visuospatial function, language and executive function) but normal or subtle functional IADL decline related to cognitive decline rather than stroke-related (4). PSD was diagnosed if at least 2 out of 4 cognitive function domains were impaired on the neuropsychologist tests with ADL impairment attributable to cognitive decline rather than stroke-related physical disability.

### Statistical Analyses

Statistical analyses were performed with SPSS 20.0 and MEDCALC^®^ 19.2.0. The Chi-squared (χ^2^) test was used to investigate differences between categorical variables among groups. Normality was tested with Shapiro-Wilk test. If variables were normally distributed, then Levene's test for homogeneity of variance was performed. Variables with homogeneity of variance, were compared using a student *t*-test or one-way analysis of variance (ANOVA) for differences between three or more groups (NC, PSCIND and PSD). If data weren't normally distributed, the Kruskal-Wallis H test was used to test for differences between the 3 diagnostic groups. Significance was set a level of 0.05. Receiver operating characteristic (ROC) curve analysis was used to assess diagnostic accuracy based on the area under the curve (AUC). ROC curves were compared using the DeLong method (30). Accuracy was excellent if AUC results were between 0.90 and 1.0, good if they were between 0.8 and 0.9, fair if between 0.7 and 0.8, poor between 0.6 and 0.7, and where values were found to be between 0.50 and 0.60 they were regarded as a fail. The optimal cut-off for each test was determined from Youden's Index (J = sensitivity + specificity−1). We estimated sample size using a precision-based calculation. Here as the expected prevalence of cognitive impairment (PSD or PSCIND) was ~70% based on existing studies (5) and the sensitivity and specificity of the Q*mci*-CN for detecting cognitive impairment is ~85% [based on existing studies of the instrument (15)], it was estimated that between 70 and 164 patients would be required at a precision of 0.1 (10%) at a significance of 0.05 (α).

## Results

In all, 230 patients with stroke were screened for the study, 125 patients with stroke were excluded as they met exclusion criteria, while 54 declined to participate. Hence, 51 patients with stroke were recruited. Among these, 17 patients did not complete the full assessment. Of the final sample of 34 patients included, 8 had normal cognition (NC), 15 were classified as having PSCIND and 11 patients were diagnosed with PSD. Patient characteristics are presented in Table 1. In all, 13 (38.2%) participants were female. The mean age of the total sample was 63 years, with a standard deviation (SD) of 13 years. The mean (±SD) age of those in the NC, PSCIND, and PSD group were 58 (± 13), 65 (±11), and 65 (±16) years, respectively. The mean (±SD) number of years in education of all participants was 13(±4) years, with a mean of 14(±5), 13(±3), and 10(±5) among the NC, PSCIND and PSD groups, respectively. There were no statistically significant differences in age (*p* = 0.338), sex (*p* = 0.254), or educational level (*p* = 0.306) between the 3 diagnostic groups. The interval between presentation with stroke and the assessment varied from 2 weeks up to 13 years.

**Table 1:** Characteristics of the participants included in total (*n* = 34) and according to diagnosis classification.

**Characteristics**	**All (*n* = 34) *N* (%) or Mean ± SD [Range]**	**NC (*n* = 8) *N* (%) or Mean ± SD [Range]**	**PSCIND (*n* = 15) *N* (%) or Mean ± SD [Range]**	**PSD (*n* = 11) *N* (%) or Mean ± SD [Range]**	***P = x***
**Gender**					
Female	13 (38.2%)	1(12.5%)	5(33.3%)	6(54.5%)	0.254
Male	21 (61.8%)	7(87.5%)	9(60%)	5(45.5%)	
Age (years)	63.38 ± 13.07 [31–85]	57.75 ± 12.78 [31–71]	65.4 ± 11.12 [45–79]	64.73 ± 15.58 [43–85]	0.338
Education (years)	12.56 ± 4.25 [0–19]	14.25 ± 4.56 [6–19]	13 ± 3.29 [6–17]	10.74 ± 4.86 [0–19]	0.306
**Living arrangements**					
Living with family	26 (76.5%)	8 (100%)	12 (80%)	8 (90.9%)	0.188
Living with a formal carer	6 (17.6%)	0 (0%)	3 (20%)	2 (18.2%)	
Living alone	2 (5.9%)	0 (0%)	0 (0%)	1 (9.1%)	
**Work intensity**					
Low	11 (32.4%)	3 (37.5%)	6 (40%)	1 (9.1%)	0.110
Medium	5 (14.7%)	3 (37.5%)	6 (40%)	7 (63.6%)	
High	5 (14.7%)	2 (25%)	2 (13.3%)	1 (9.1%)	
Other (none, volunteer or not provided)	13 (38.2%)	0 (0%)	1 (6.7%)	2 (18.2%)	
Hypertension (proportion with)	20 (58.8%)	4 (50%)	12 (80%)	10 (90.0%)	0.021^*^
Hyperglycaemia (proportion with)	13 (38.2%)	3 (37.5%)	5 (33.3%)	4 (36.4%)	0.204
Hyperlipemia (proportion with)	9 (26.5%)	1 (12.5%)	8 (53.3%)	1 (9.1%)	0.034^*^
Dyssomnia (proportion with)	7 (20.6%)	2 (25%)	4 (26.7%)	1 (9.1%)	0.202

*NC, normal cognitive; PSCIND, post-stroke cognitive impairment no dementia; PSD, post-stroke dementia; SD, standard deviation*.

* *Statistically significant (p < 0.05)*.

### Cognitive Test Scoring and Administration

The median scores and administration times for each diagnostic group with their interquartile range (IQR) including comparisons between all 3 diagnostic groups and pair-wise comparisons are presented in Table 2. We found statistically significant differences in total median test scores between all 2 diagnostic groups (NC, PSCIND, and PSD) (*p* < 0.01). Analyses showed that all 3 diagnostic groups were different from each other, and higher tests scores related with higher level of cognitive ability. While the MoCA showed a clear gradient in median scores from NC to PSCIND and PSD (decreasing from 27 to 22 to 13), the Q*mci*-CN had similar median scores for NC and PSCIND. The MMSE-CN had similar values for PSCIND and PSD. Comparisons between administration times for the cognitive screens are presented in Table 3. The median (± IQR) administration time for the Q*mci*-CN was 328.50 ± 50.25 s vs. 610.87 ± 116.75 for the MoCA-CN, a statistically significant difference (*p* < 0.0001). The median administration time for the MMSE-CN was 280.17 ± 43.75, which was also shorter than the MoCA-CN (*p* < 0.0001). Examining administration times by diagnostic classification group showed that there were no significant differences for either the Q*mci*-CN (*p* = 0.144), MoCA-CN (*p* = 0.333), or MMSE-CN (*p* = 0.173). A moderate gradient effect was seen with the Q*mci*-CN (*r* = 0.52, *p* = 0.771); those with better cognition had higher scores, albeit these were not statistically significantly shorter.

**Table 2:** Test scores and administration times for the Chinese versions of the quick mild cognitive impairment screen (Q*mci*-CN), montreal cognitive assessment (MoCA-CN) and mini mental state examination (MMSE-CN) by diagnostic group.

**Cognitive test (score/administration time)**	**Total sample (*n* = 34)**	**NC (*n* = 8)**	**PSCIND (*n* = 15)**	**PSD (*n* = 11)**	**Kruskal–Wallis H-test comparing all 3 groups (*P* = x)**	**NC vs. PSCIND (*P* = x)**	**NC vs. Post-stroke CI (PSCIND & PSD) (*P* = x)**	**PSCIND vs. PSD (*P* = x)**
Q*mci*-CN scores (median ± IQR)	55.50 ± 14.25 [0–69]	57.50 ± 8.25 [48–66]	57.00 ± 7.00 [47–69]	37.00 ± 35.00 [4–57]	*P* < 0.001^*^	*P* = 0.548	*P* = 0.074	*P* < 0.001^*^
MoCA-CN scores (median ± IQR)	22.00 ± 8.50 [1–30]	27.00 ± 5.50 [22–30]	22.00 ± 3.00 [15–27]	13.00 ± 9.00 [1–22]	*P* < 0.001^*^	*P* = 0.007^*^	*P* < 0.001^*^	*P* < 0.001^*^
MMSE-CN scores (median ± IQR)	25.00 ± 3.79	27.00 ± 1.00	24.00 ± 3.71	23.00 ± 17.00	*P* = 0.001^*^	*P* = 0.040	*P* = 0.003^*^	*P* = 0.011
	[0–30]	[25–29]	[23–30]	[0–25]				
Q*mci*-CN time (s) (median ± IQR)	328.50 ± 50.25 [150–408]	273.00 ± 91.00 [230–382]	331.00 ± 41.00 [298–406]	335.00 ± 45.00 [150–408]	*P* = 0.144	*P* = 0.034	*P* = 0.082	*P* = 0.878
MoCA-CN time (s) (median ± IQR)	610.87 ± 116.75 [176–1023]	580.50 ± 154.65 [357–633]	617.00 ± 186.00 [454–791]	610.87 ± 286.00 [176–1023]	*P* = 0.333	*P* = 0.076	*P* = 0.110	*P* = 1.000
MMSE-CN time(s) (median ± IQR)	280.17 ± 43.75	255.00 ± 59.38	280.17 ± 15.00	280.17 ± 70.00	*P* = 0.173	*P* = 0.076	*P* = 0.110	*P* = 0.799
	[178–510]	[178–326]	[193–329]	[182–510]				

*CI, cognitive impairment; IQR, interquartile range; NC, normal cognition; PSCIND, post-stroke cognitive impairment no dementia; PSD, post-stroke dementia*.

* *Statistically significant (p < 0.01)*.

**Table 3:** ANOVA *Post-hoc* pair-wise analysis comparing administration times for the Q*mci*-CN screen, MoCA-CN, and MMSE-CN.

**Variable**	**Group 1**	**Group 2**	**Mean difference**	**Std. error**	***P*-value**	**95% Confidence interval**	
						**Lower bound**	**Upper bound**
Time taken	Q*mci*-CN	MoCA-CN	−291.753	30.567	<0.0001	−368.33	−215.18
	Q*mci*-CN	MMSE-CN	38.951	18.562	0.123	−7.48	85.38
	MoCA-CN	MMSE-CN	330.704	33.434	<0.0001	247.84	413.57

*MMSE-CN, mini mental state examination; MoCA-CN, montreal cognitive assessment; Qmci-CN, quick mild cognitive impairment screen*.

### Screening for Post-stroke Cognitive Impairment (PSCIND and PSD)

Measures of diagnostic accuracy including optimal cut-offs for each instrument are presented in Table 4. Cross cross-tabulated results are provided as Supplementary Material. Comparisons between instruments are presented in Table 5. ROC curve analyses showed that the Q*mci*-CN, MoCA-CN and MMSE-CN had similar accuracy in separating PSD from NC; the MoCA-CN (AUC 0.994) had slightly higher accuracy but this was not statistically greater than the Q*mci*-CN (AUC 0.938) or MMSE-CN (AUC 0.983). At their optimal cut-offs, all 3 tests showed excellent sensitivity but poor specificity for the ability to separate PSD from NC. The Q*mci*-CN had an optimal cut-off score of ≤47 vs. ≤19.5 for the MoCA-CN and ≤26 for the MMSE-CN. The results also showed that the Q*mci*-CN, MMSE-CN, and MoCA-CN had statistically similar accuracy in separating PSCIND from PSD. The Q*mci*-CN had an AUC of 0.906 compared to an AUC of 0.936 for the MoCA-CN and an AUC of 0.791 for the MMSE-CN.

**Table 4:** Area Under the Curve (AUC) values and optimal cut-offs for the Chinese versions of the quick mild cognitive impairment (Q*mci*-CN) screen, montreal cognitive assessment (MoCA-CN), and mini mental state examination (MMSE-CN).

**Diagnostic classification**	**Cognitive screen**	**AUC [95% CI]**	**Optimal cut-off point (youden index)**	**Sensitivity and specificity [%]**
PSCIND vs. NC	Q*mci*-CN	0.583 [0.336–0.831]	≤55.5	Sensitivity = 88%, Specificity = 53%
	MoCA-CN	0.842 [0.672–1.000]	≤26	Sensitivity = 63%, Specificity = 7%
	MMSE-CN	0.763 [0.561–0.964]	≤26.5	Sensitivity = 88%, Specificity = 27%
PSD vs. NC	Q*mci*-CN	0.938 [0.833–1.000]	≤47	Sensitivity = 100%, Specificity = 18%
	MoCA-CN	0.994 [0.972–1.000]	≤19.5	Sensitivity = 100%, Specificity = 9%
	MMSE-CN	0.983 [0.935–1.000]	≤26	Sensitivity = 88%, Specificity = 0%
PSD vs. PSCIND	Q*mci*-CN	0.909 [0.783–1.000]	≤46.5	Sensitivity = 100%, Specificity = 18%
	MoCA-CN	0.936 [0.839–1.000]	≤18.5	Sensitivity = 93%, Specificity = 9%
	MMSE-CN	0.791 [0.613–0.969]	≤21	Sensitivity = 100%, Specificity = 55%
Post stroke CI (PSCIND/PSD) vs. NC	Q*mci*-CN	0.733 [0.558–0.909]	≤55.5	Sensitivity = 88%, Specificity = 39%
	MoCA-CN	0.906 [0.800–1.000]	≤21.5	Sensitivity = 100%, Specificity = 39%
	MMSE-CN	0.856 [0.727–0.984]	≤26.5	Sensitivity = 88%, Specificity = 15%
PSD vs. Post stroke CI (NC/PSCIND)	Q*mci*-CN	0.919 [0.808–1.000]	≤46.5	Sensitivity = 100%, Specificity = 18%
	MoCA-CN	0.957 [0.889–1.000]	≤18.5	Sensitivity = 96%, Specificity = 9%
	MMSE-CN	0.858 [0.730–0.985]	≤23.64	Sensitivity = 74%, Specificity = 27%

*CI, cognitive impairment; NC, normal cognition; PSCIND, post-stroke cognitive impairment no dementia; PSD, post stroke dementia*.

**Table 5:** Pair-wise comparisons between the diagnostic accuracy of the Chinese versions of the quick mild cognitive impairment (Q*mci*-CN) screen, montreal cognitive assessment (MoCA-CN) and mini mental state examination (MMSE-CN).

**Diagnostic classification**	**Comparison group (area under the curve)**		**Z-statistic**	***P*-value**
PSCIND vs. NC	Q*mci*-CN	MoCA-CN	2.442	0.015^*^
	(0.583)	(0.842)		
	Q*mci*-CN	MMSE-CN	1.457	0.145
	(0.583)	(0.763)		
	MoCA-CN	MMSE-CN	0.747	0.455
	(0.842)	(0.763)		
PSD vs. NC	Q*mci*-CN	MoCA-CN	0.517	0.237
	(0.938)	(0.994)		
	Q*mci*-CN	MMSE-CN	1.818	0.293
	(0.938)	(0.983)		
	MoCA-CN	MMSE-CN	1.866	0.406
	(0.994)	(0.983)		
PSD vs. PSCIND	Q*mci*-CN	MoCA-CN	0.517	0.605
	(0.909)	(0.936)		
	Q*mci*-CN	MMSE-CN	1.818	0.069
	(0.909)	(0.791)		
	MoCA-CN	MMSE-CN	1.866	0.062
	(0.936)	(0.791)		
Post stroke CI (PSCIND/PSD) vs. NC	Q*mci*-CN	MoCA-CN	2.489	0.013^*^
	(0.733)	(0.906)		
	Q*mci*-CN	MMSE-CN	1.594	0.111
	(0.733)	(0.856)		
	MoCA-CN	MMSE-CN	0.842	0.400
	(0.906)	(0.856)		
PSD vs. NC/PSCIND	Q*mci*-CN	MoCA-CN	0.795	0.426
	(0.919)	(0.957)		
	Q*mci*-CN	MMSE-CN	1.216	0.224
	(0.919)	(0.858)		
	MoCA-CN	MMSE-CN	1.821	0.069
	(0.957)	(0.858)		

*CI, cognitive impairment; NC, normal cognition; PSCIND, post-stroke cognitive impairment no dementia; PSD, post-stroke dementia*.

* *Statistically Significant*.

**Figure 1:**
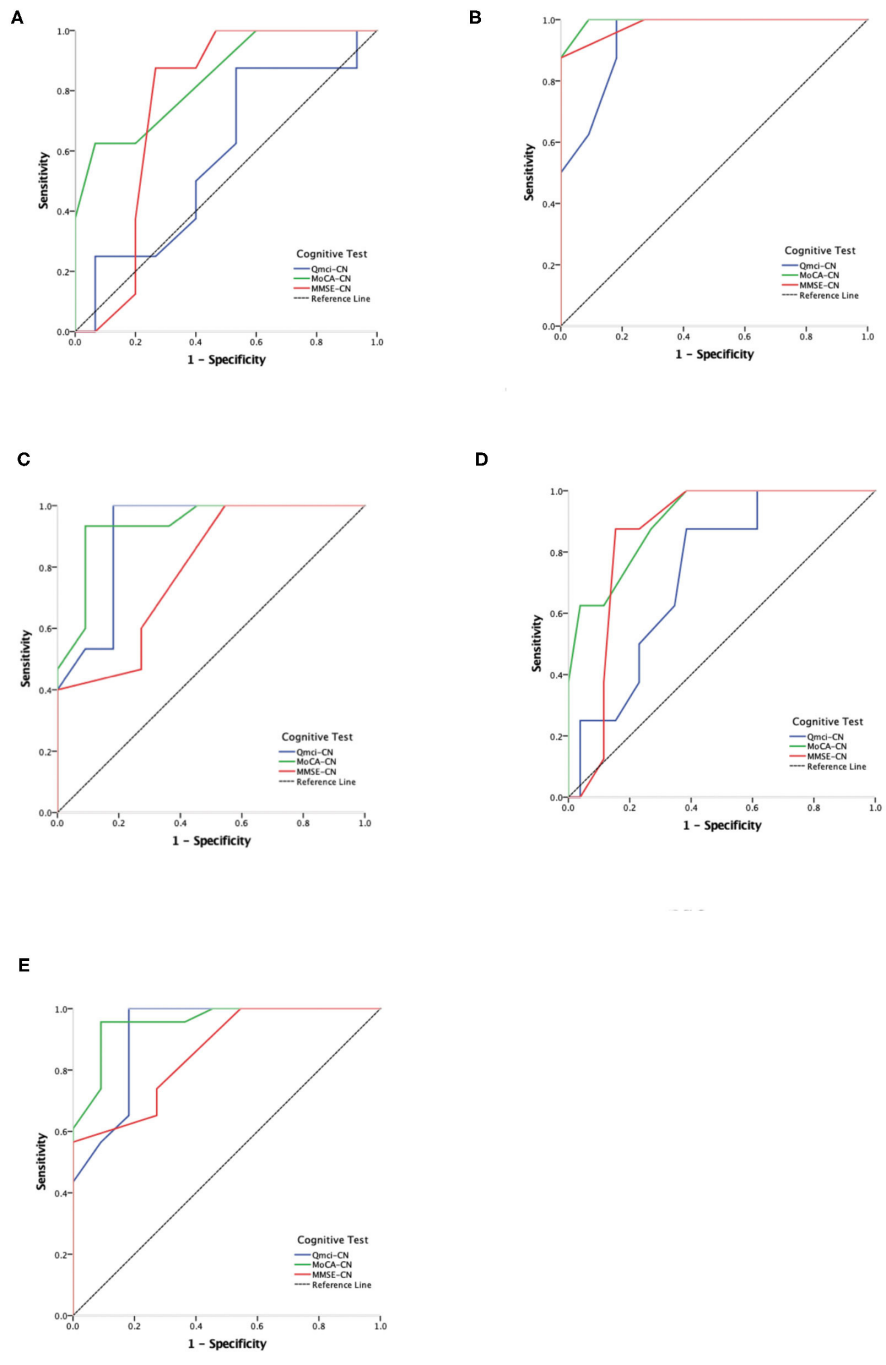
Receiver Operating Characteristic (ROC) curve analysis comparing the Chinese versions of the Quick Mild Cognitive Impairment (Q*mci*-CN) screen, Montreal Cognitive Assessment (MoCA-CN) and Mini Mental State Examination (MMSE-CN) in separating normal cognition (NC), post-stroke cognitive impairment no dementia (PSCIND) and post-stroke dementia (PSD). **(A)** NC vs. PSCIND; **(B)** NC vs. PSD; **(C)** PSCIND vs. PSD; **(D)** NC vs. PSCIND/PSD; **(E)** PSD vs. NC/PSCIND.

ROC analysis showed that the Q*mci*-CN had an AUC of 0.733 compared with AUCs of 0.906 and 0.856 for the MoCA-CN and MMSE-CN, respectively, in separating PSCI (either PSCIND/PSD) from NC. The Q*mci*-CN was less accrate than the MoCA-CN (*p* = 0.013) but was similar to the MMSE-CN (*p* = 0.11) in differentiating PSCI (either PSCIND/PSD) from NC. The Q*mci*-CN was also less accurate in its ability to distinguish PSCIND from NC (AUC 0.583) compared with the MoCA-CN (AUC of 0.842), (*p* = 0.015). ROC curves are presented in Figure 1.

## Discussion

This study compared the diagnostic accuracy of the newly-developed Q*mci*-CN for its ability to screen for PSCI with the widely-used MoCA-CN and MMSE-CN in a rehabilitation population in China. This is, to our knowledge, the first study validating the Q*mci* screen in a post-stroke population in any language. The results showed that all three of these scales are equally able to differentiate PSD from NC and PSD from PSCIND. The Q*mci*-CN had excellent accuracy (AUC > 0.90) in separating both diagnostic groups but poor accuracy in distinguishing PSCIND from NC. While the MoCA-CN was significantly more accurate in separating post-stroke CI (either PSCIND or PSD) from normal and in turn PSCIND from NC than the Q*mci*-CN, it had a statistically significantly longer median administration time. The MoCA-CN took almost twice as long to score. This is a consistent finding in studies comparing the two instruments (15). It also had similar accuracy to the MMSE-CN but again longer administration times. The Q*mci*-CN and MMSE-CN had similar administration times in this study.

These results are different from previous research examining the diagnostic accuracy of other language versions of the Q*mci*, especially the English version, which show that the Q*mci* screen is more accurate in discriminating MCI (equivalent of PSCIND) from both normal controls and dementia than the MoCA and the MMSE (17–20). These studies also generally show that the MMSE is less accurate than both the MoCA and Q*mci* screen (15). The reasons for why this study differs from others is most likely related to its very small sample size. Sample size calculations based on previous studies suggest that this study was not powered adequately to show superiority of one test over the other. The sample size in some of the diagnostic groups, particularly the NC group (*N* = 8), is too small to interpret the findings reliably. Further, those included in this study were a different cohort with all patients recovering from an acute stroke. These may have cognitive impairment in multiple other cognitive domains, while retaining memory (31). The Q*mci*-CN is better able to detect amnestic type cognitive impairment (32) compared with other short screens as it is more heavily weighted toward memory. This said, the goal of this study was not to show superiority of the Q*mci*-CN but to examine its utility and psychometric properties in patients post-stroke compared with more-commonly used screening tests.

This study also examines the optimal cut-off points for the 3 instruments to identify NC, PSCIND, and PSD. At the established cut-off for cognitive impairment (≤26) rather than post-stroke cognitive impairment (≤22) (33), the MoCA had excellent sensitivity but poor specificity. While the MMSE may lack sensitivity for single domain cognitive impairment (33), using the optimal cut-off points identified using Youden's Index, this study found that the MMSE had a similarly high sensitivity but low specificity. The optimal cut-off point for the Q*mci*-CN in separating PSCIND from NC was ≤55.5 compared with ≤47 to differentiate PSD from normal. While lower than the results in Irish (20) and Canadian (34) cohorts in patients attending memory clinics, these are more similar to results in Turkey (<53 for separating MCI from normal cognition) (22, 35) and recently in Greece (<51 for separating MCI from normal cognition) (36). Possible reasons for this difference might include the background of participants including their educational level, albeit the sample included here had a similar median number of years in education to those in North America and Western Europe and higher than those in Turkey and Greece. Again the setting may have influenced the cut-offs as all patients here were recruited from rehabilitation clinics having had a recent acute stroke. This may have influenced the performance of patients in testing across several cognitive domains resulting in lower total scores, irrespective of diagnostic classification. However, given that this is a small sample, care should be taken when interpreting and applying these cut-offs. The authors caution that normative data are required to develop accurate cut-off scores for the Q*mci*-CN and to determine if these are comparable with the English language version of the Q*mci* screen.

## Limitations

This study has a number of limitations as follows. First, the definitions of PSCI in the literature and in clinical practice are variable, differing from study to study (37). In our research, we defined PSCI as a post-stroke neuropsychological syndrome with any cognitive impairment, which develops following a stroke event, including the subgroups of PSD and PSCIND. This definition does not suggest any particular underlying neuropathological process. Other definitions include time limits on the onset of cognitive decline, though we did not apply this. This is a limitation. For example, a national Korean study examining the epidemiology of vascular cognitive defined PSD as any major cognitive impairment evident more than 3 months after a stroke (38). Others suggest the final diagnosis of PSD should be delayed until at least 6 months as stroke commonly results in delirium or transient reductions in cognition that do not persist. Hence, making the diagnosis too early may result in a higher prevalence of PSCI than would be expected (39). Here no time interval was pre-specified and we recruited patients having completed a stroke at least 2 weeks but up to 13 years after the event. Hence, to ensure homogeneity and comparability, further study applying pre-specified time-based definitions of PSCI are therefore needed. Second, as the diagnostic criteria for PSCI incorporates ADL function and many patients with stoke have related physical impairment, it can be difficult to assess whether the changes in ADLs are truly related to cognitive impairment (37). This may increase the heterogeneity among the 3 groups and is a potential confounder. Thirdly, as patients were all recruited from the department of rehabilitation medicine, most of these may have completed cognitive assessment before, with either the MoCA-CN and the MMSE-CN, potentially creating learning effects and introducing bias. This said, none of the patients would have been tested with the Q*mci*-CN while inpatients. Finally, the sample here is small and likely prone to selection bias, reducing the generalisability of the results and under-powering the study to show superiority of one screening instrument over another based on our sample size calculation, increasing the chance of type II errors. Only those who received rehabilitation were available and included, potentially reducing the generalisability of the findings. Hence, further studies examining these instruments in a broader sample of patients post-stroke including those who did not receive formal rehabilitation or were unlikely to benefit from rehabilitation should be recruited. As the sample size was small, recruitment in multiple sites over a longer period of time is also needed to increase the reliability, generalisability and ability to confirm superiority of one instrument over another.

## Conclusion

In conclusion, the Q*mci*-CN was accurate in screening for PSD in a post-stroke rehabilitation clinic and had excellent accuracy in separating PSD from PSCIND and PSD from NC. The administration time of the Q*mci*-CN was significantly lower than the MoCA-CN, suggesting it is a reasonable alternative in busy clinics. However, the Q*mci*-CN had poor accuracy in separating PSCIND from NC and PSCI from NC, suggesting it may lack accuracy for certain subgroups even if its administration time is shorter. The MoCA-CN and MMSE-CN both had similar accuracy in PSCI, which given the shorter administration time of the MMSE-CN suggests that it may be the better instrument to use in this setting. However, because this was a small study, under-powered to show superiority of one instrument over another, with several limitations, further study is now needed before recommending the routine use of the Q*mci*-CN in order to confirm these findings by recruiting a larger sample size and externally validating the Q*mci*-CN in other samples and populations.

## Data Availability Statement

The raw data supporting the conclusions of this article will be made available by the authors, without undue reservation.

## Ethics Statement

The studies involving human participants were reviewed and approved by this study received ethical approval from The Sixth Affiliated Hospital of Sun Yat-sen University, reference number 2019ZSLYEC-110. The patients/participants provided their written informed consent to participate in this study.

## Author Contributions

YX and LY: design, concept, data collection, analysis, writing, and revising manuscript. YW: design, concept, and supervision. YL, SP, WW, WL, PC, WZ, YD, SG, and LS: concept and data collection. YX and LY: statistical analysis and supervision of statistics. DM: design and concept. RO'C: design, concept, analysis, writing, and revising manuscript. All authors contributed to the article and approved the submitted version.

## Conflict of Interest

DM and RO'C are co-copyright holders of the Q*mci* screen. DM, RO'C, and YX are copyright holders of the translation into Chinese, the Q*mci*-CN screen. The remaining authors declare that the research was conducted in the absence of any commercial or financial relationships that could be construed as a potential conflict of interest.
