# Supplementary material for: Screening for Cognitive Impairment After Stroke: Validation of the Chinese Version of the Quick Mild Cognitive Impairment Screen
Source: Front Neurol. 2021 Mar 5;12:608188. doi: 10.3389/fneur.2021.608188 (PMC7982853; doi:10.3389/fneur.2021.608188)
Supplement: Supplementary file 1 [file Data_Sheet_1.docx]

Supplement material: cross-tabulation results

1.Q*mci*-CN

| PSCIND vs NC (Qmci-CN) | | | |
| --- | --- | --- | --- |
| ≤ 55.5 |  |  |  |
|  | PSCIND (1) | NC (0) | SUM |
| Qmci-CN pos at ≤55.5 (1) | 7 | 1 | 8 |
| Qmci-CN for CI >55.5 (0) | 8 | 7 | 15 |
|  | 15 | 8 | 23 |

| PSD vs NC (Qmci-CN) | | | |
| --- | --- | --- | --- |
| ≤ 47 |  |  |  |
|  | PSD (1) | NC (0) | SUM |
| Qmci-CN pos at ≤47 (1) | 9 | 0 | 9 |
| Qmci-CN for CI >47 (0) | 2 | 8 | 10 |
|  | 11 | 8 | 19 |

| PSD vs PSCIND (Qmci-CN) | | | |
| --- | --- | --- | --- |
| ≤ 46.5 |  |  |  |
|  | PSD (1) | PSCIND (0) | SUM |
| Qmci-CN pos at ≤46.5 (1) | 9 | 0 | 9 |
| Qmci-CN for CI >46.5 (0) | 2 | 15 | 17 |
|  | 11 | 15 | 26 |

| PSCI vs NC (Qmci-CN) | | | |
| --- | --- | --- | --- |
| ≤ 55.5 |  |  |  |
|  | PSCI (1) | NC (0) | SUM |
| Qmci-CN pos at ≤55.5 (1) | 16 | 1 | 17 |
| Qmci-CN for CI >55.5 (0) | 10 | 7 | 17 |
|  | 26 | 8 | 34 |

| PSD vs PSCI (Qmci-CN) | | | |
| --- | --- | --- | --- |
| ≤ 46.5 |  |  |  |
|  | PSD (1) | PSCI (0) | SUM |
| Qmci-CN pos at ≤46.5 (1) | 9 | 0 | 9 |
| Qmci-CN for CI >46.5 (0) | 2 | 23 | 25 |
|  | 11 | 23 | 34 |

2. MoCA-CN

| PSCIND vs NC (MoCA-CN) | | | |
| --- | --- | --- | --- |
| ≤ 26 |  |  |  |
|  | PSCIND(1) | NC (0) | SUM |
| MoCA -CN pos at ≤26 (1) | 11 | 1 | 12 |
| MoCA -CN for CI >26 (0) | 4 | 7 | 11 |
|  | 15 | 8 | 23 |

| PSD vs NC (MoCA-CN) | | | |
| --- | --- | --- | --- |
| ≤ 19.5 |  |  |  |
|  | PSD (1) | NC (0) | SUM |
| MoCA -CN pos at ≤19.5 (1) | 5 | 0 | 5 |
| MoCA -CN for CI >19.5 (0) | 6 | 8 | 14 |
|  | 11 | 8 | 19 |

| PSD vs PSCID (MoCA-CN) | | | |
| --- | --- | --- | --- |
| ≤ 18.5 |  |  |  |
|  | PSD (1) | PSCID  (0) | SUM |
| MoCA -CN pos at ≤18.5 (1) | 4 | 0 | 4 |
| MoCA -CN for CI >18.5 (0) | 7 | 15 | 22 |
|  | 11 | 15 | 26 |

| PSCI vs NC (MoCA-CN) | | | |
| --- | --- | --- | --- |
| ≤ 21.5 |  |  |  |
|  | PSCI (1) | NC (0) | SUM |
| MoCA -CN pos at ≤21.5 (1) | 5 | 0 | 5 |
| MoCA -CN for CI >21.5 (0) | 21 | 8 | 29 |
|  | 26 | 8 | 34 |

| PSD vs PSCI (MoCA-CN) | | | |
| --- | --- | --- | --- |
| ≤ 18.5 |  |  |  |
|  | PSD (1) | PSCI (0) | SUM |
| MoCA -CN pos at ≤18.5 (1) | 4 | 0 | 4 |
| MoCA -CN for CI >18.5 (0) | 7 | 23 | 30 |
|  | 11 | 23 | 34 |

3. MMSE-CN

| PSCIND vs NC (MMSE-CN) | | | |
| --- | --- | --- | --- |
| ≤ 26.5 |  |  |  |
|  | PSCIND(1) | NC (0) | SUM |
| MMSE -CN pos at ≤26.5 (1) | 14 | 3 | 17 |
| MMSE -CN for CI >26.5 (0) | 1 | 5 | 6 |
|  | 15 | 8 | 23 |

| PSD vs NC (MMSE-CN) | | | |
| --- | --- | --- | --- |
| ≤ 26 |  |  |  |
|  | PSD (1) | NC (0) | SUM |
| MMSE -CN pos at ≤26 (1) | 11 | 3 | 14 |
| MMSE -CN for CI >26 (0) | 0 | 5 | 5 |
|  | 11 | 8 | 19 |

| PSD vs PSCIND (MMSE-CN) | | | |
| --- | --- | --- | --- |
| ≤ 21 |  |  |  |
|  | PSD (1) | PSCIND(0) | SUM |
| MMSE -CN pos at ≤21 (1) | 10 | 5 | 15 |
| MMSE -CN for CI >21 (0) | 1 | 10 | 11 |
|  | 11 | 15 | 26 |

| PSCI vs NC (MMSE-CN) | | | |
| --- | --- | --- | --- |
| ≤ 26.5 |  |  |  |
|  | PSCI (1) | NC (0) | SUM |
| MMSE -CN pos at ≤26.5 (1) | 25 | 3 | 28 |
| MMSE -CN for CI >26.5 (0) | 1 | 5 | 6 |
|  | 26 | 8 | 34 |

| PSD vs PSCI (MoCA-CN) | | | |
| --- | --- | --- | --- |
| ≤ 23.64 |  |  |  |
|  | PSD (1) | PSCI(0) | SUM |
| MMSE -CN pos at ≤23.64 (1) | 11 | 13 | 24 |
| MMSE -CN for CI >23.64(0) | 0 | 5 | 5 |
|  | 11 | 18 | 29 |
